# Age Estimation Using the Pulp-to-Tooth Area Ratio in an Indian Population Using Cone-Beam Computerised Tomography (CBCT): A Retrospective Study

**DOI:** 10.7759/cureus.65287

**Published:** 2024-07-24

**Authors:** Pratiksha Zambare, Nupura Vibhute, Uzma Belgaumi, Vidya Kadashetti, Rashmi Gangavati, Wasim Kamate

**Affiliations:** 1 Department of Oral Pathology and Microbiology, School of Dental Sciences, Krishna Vishwa Vidyapeeth (Deemed to be University), Karad, IND

**Keywords:** pulp-to-tooth area ratio (ptar), two-dimensional (2d), three-dimensional (3d), computed tomography (ct), cone-beam computed tomography (cbct)

## Abstract

Background: Age estimation, whether for living or deceased individuals, is crucial in forensic sciences. Traditionally, the pulp-to-tooth area ratio determined from periapical radiographs has been employed as a non-invasive method for estimating age. Cone-beam computed tomography (CBCT) represents a newer technique, providing three-dimensional images of teeth in living individuals.

Aim: This study aims to estimate age using the pulp-to-tooth area ratio of four teeth (the permanent maxillary central incisor, lateral incisor, canine, and first premolar.

Study design: The study included ninety subjects ranging in age from 18 to 70 years.

Conclusion: This study concluded that the correlation between actual age and estimated age varied across different tooth types. There was a strong positive correlation between the maxillary right central incisor and maxillary right canine tooth, while the maxillary left canine tooth exhibited a slightly weaker but still positive correlation. This indicates that the maxillary right central incisor and maxillary right canine tooth are the most reliable for estimating age. Additionally, the study found that the linear regression results for estimating age based on the pulp-to-tooth area ratio, categorised by sex and each type of tooth, showed strong associations between age and the ratio for the maxillary left central incisor and maxillary left canine teeth in males, and for the maxillary left central incisor in females.

## Introduction

"Forensic science offers great potential, as it draws on almost every discipline and, in doing so, creates widespread opportunity for innovation." - Mark Walport

Age estimation is vital in forensic medicine and dentistry for identifying deceased individuals and investigating crimes and accidents. Accurately determining age is essential for establishing a person's identity. This practice is employed by anthropologists, archaeologists, and forensic scientists [1]. Furthermore, age changes are significant in crime investigations. Age estimation can determine the appropriate punishment for an accused, particularly when their legal adult status is in question. It is also crucial for immigrants without definitive age and birth records, and for determining the legal status of individuals like runaway brides. Thus, age estimation applies to both living and deceased subjects across various age groups, including children, adolescents, and adults [2].

Various parts of the human body can be utilised for age estimation. However, in cases of severe accidents, burns, or buried remains, many body parts may lose their natural form and become unsuitable for age estimation [3]. Teeth can persist for many years after death, making them highly suitable for human age estimation. Additionally, teeth are minimally impacted by environmental factors. As the hardest part of the human body, teeth consist of enamel, dentin, cementum and pulp. Assessing morphological changes in teeth generally requires sectioning, which is not feasible for living individuals. In adults, dental age prediction relies on quantifying age-related changes in teeth, such as secondary dentin deposition. This can be accurately achieved through histological and biochemical methods. However, these techniques necessitate tooth extraction and often sectioning or processing, which may not be practical for living adults or permitted in certain jurisdictions that restrict tissue collection from human remains. Consequently, non-invasive methods that do not involve tooth extraction or sectioning are preferred for age evaluation. Therefore, age estimation methods primarily rely on radiographic imaging [2]. In 1995, Kvaal et al. developed a method for age estimation based on measuring the pulp dimensions resulting from the deposition of secondary dentin [4]. Their findings indicated a strong correlation between pulp width and ageing [4].

The initial application of radiographic techniques in dental identification dates back to Schuller in 1921 [5]. The implementation of age estimation has required the use of this technology for over half a century [5]. Kvaal et al. were among the early proponents of using radiographs for dental age estimation in adults [4]. They suggested various measurements of tooth length, width, and pulp dimensions [4]. Similarly, Cameriere et al. introduced a method for age estimation that focused on two-dimensional measurements, specifically the tooth and pulp area [6].

Panoramic and periapical radiography can be utilised to measure the pulp-to-tooth ratio. However, these methods have limitations, including the two-dimensional (2D) nature of the images, image magnification, and distortion [3]. Currently, three-dimensional (3D) imaging techniques, such as cone-beam computed tomography (CBCT), provide valuable 3D information about teeth, enabling more accurate measurement of tooth and pulp dimensions compared to 2D radiography. CBCT is considered the most ideal and accurate method for measuring the pulp-to-tooth ratio [7].

Understanding the importance of secondary dentin deposition, the current study aims to estimate age by using the pulp-to-tooth area ratio measured with CBCT for four teeth: the permanent maxillary central incisor, lateral incisor, canine, and first premolar.

## Materials and methods

The present study was a descriptive observational study involving the analysis of 360 CBCT scans from outpatients visiting the School of Dental Sciences, Krishna Vishwa Vidyapeeth, Karad.

Inclusion criteria

The patients' data included individuals aged 18 to 70 years. The study focused on fully erupted teeth (central incisor, lateral incisor, canine, and first premolar) in either the right or left maxillary quadrant. High-quality images were required, and only teeth with fully developed roots were considered.

Exclusion criteria

The study excluded teeth with dental caries, periapical pathologies, severe attrition, abrasion, erosion, or any developmental anomalies. Teeth that had undergone endodontic treatment or showed signs of restorations and crowns were also excluded. Incompletely erupted teeth, those with pulp stones or resorption, prosthetically rehabilitated teeth, and those affected by occlusal trauma were not considered. Additionally, any history of surgery or trauma in the craniofacial region led to exclusion from the study.

Ninety patients reporting to the School of Dental Sciences, Krishna Vishwa Vidyapeeth, Karad, who met the inclusion criteria, were enrolled in the current study.

Ethical clearance

Ethical clearance (protocol number: 01512022'2023) was obtained from the Institutional Ethics Committee of Krishna Vishwa Vidyapeeth, Karad, before the commencement of the study. Permission was also secured from the Department of Oral Pathology and Microbiology and the Department of Oral Medicine, Diagnosis & Radiology of the School of Dental Sciences, Krishna Vishwa Vidyapeeth, Karad.

A patient information sheet, available in both Marathi and English, was provided to each patient. This sheet detailed the procedure to be performed, the purpose of the study, confidentiality assurances, risks, volunteer participation, benefits, and the absence of disadvantages should they choose not to participate. All participants were given a thorough explanation of these points. Before starting the study, written informed consent was obtained from each participant.

Sample size

The sample size (n) was derived by using the “comparing two means” formula:

 n = (Zα/2 + Zβ)2 * 2 * σ2/d2

where "Zα/2" is the critical value at a confidence level of 95%; "α" is 0.05 and the critical value was 1.96; "Zβ" is for a power of 80%; "β" is 0.2 and the critical value was 0.84; "σ2" is the population variance; and "d2" is the mean difference between the two groups. The mean of the first group is 0.76 and the mean of the second group is 0.65.

Substituting the values in the above formula, a total sample size of 90 patients was derived.

Study design

The present study was descriptive and retrospective.

Methodology

**Figure 1:**
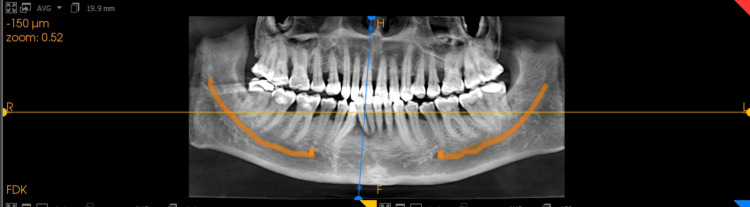
Obtaining the mid-sagittal segment along the tooth's long axis through manual curved slicing.

**Figure 2:**
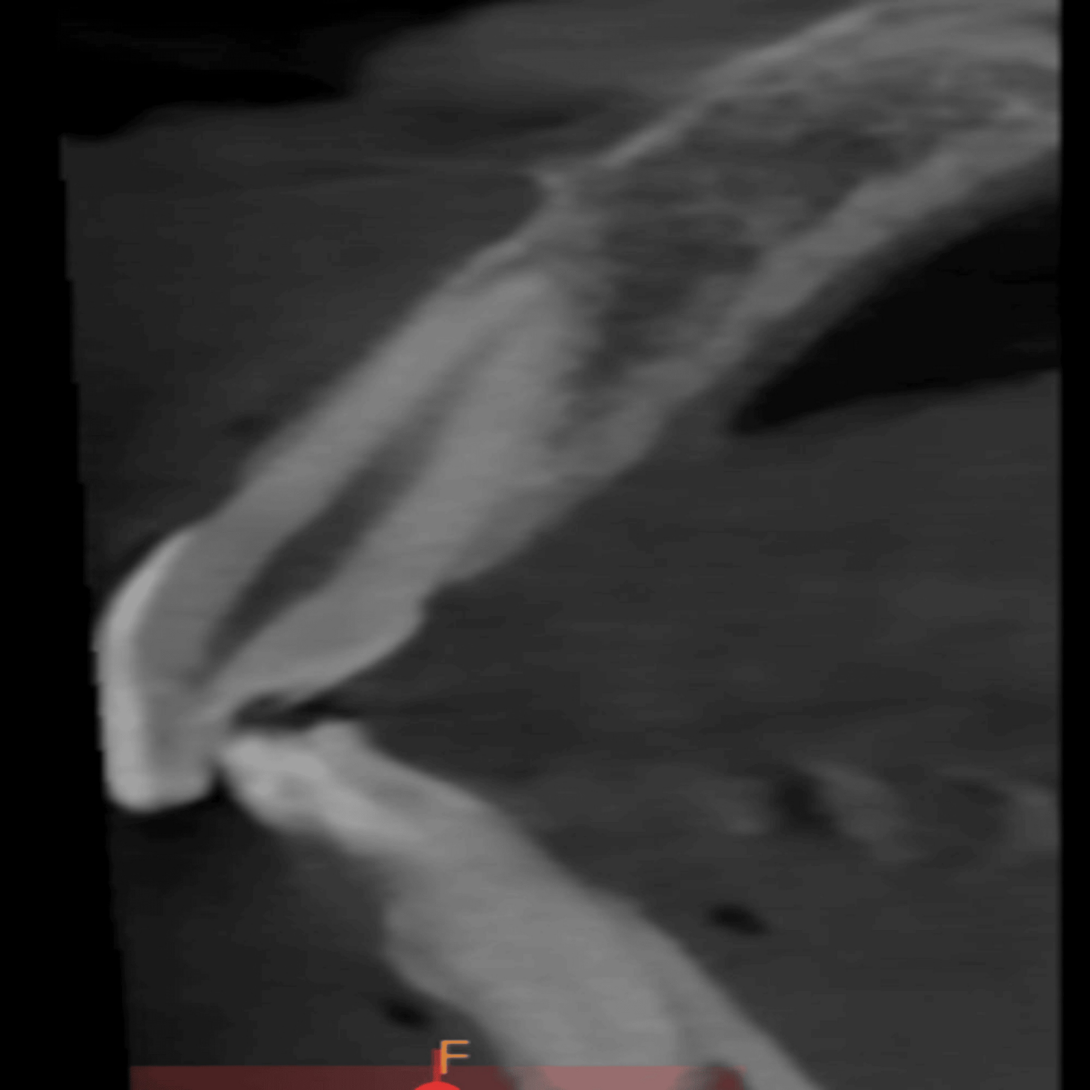
Mid-sagittal section of the maxillary central incisor tooth being examined for analysis.

CBCT scans from the Department of Oral Medicine, Diagnosis & Radiology, School of Dental Sciences, Krishna Vishwa Vidyapeeth, Karad were utilised in this study. These scans were obtained using the Cone Beam 3D Dental Imaging System, version 3.1.62, (Imaging Sciences International, Hatfield, PA, USA). The scans had exposure parameters of 120 kV and 18 mA and were taken with a voxel size of 0.30 mm and a scanning time of 20 seconds. The necessary data was selected and saved on an external CD drive for this study. The CBCT data was in DICOM (Digital Imaging and Communications in Medicine) format and was accessed using KODAK Dental Imaging Software 6.8 (Care Stream Health Inc., New York, USA). Manual curved slicing was performed on the sagittal section to obtain the mid-sagittal segment along the tooth's long axis (Figures 1, 2).

**Figure 3:**
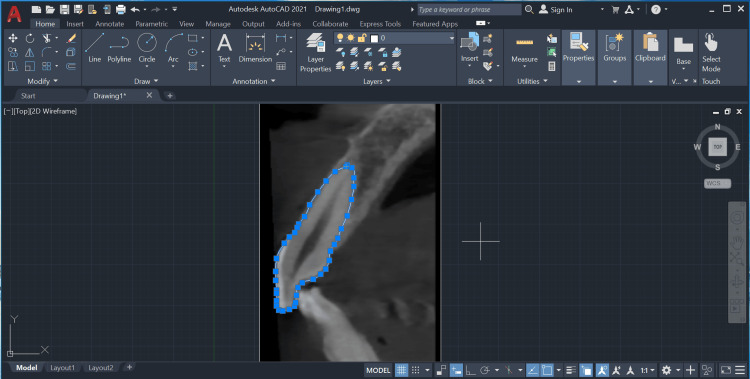
Points on the tooth outline are marked using the point tool on AutoCAD's Draw Toolbox.

These images were exported as high-resolution JPEG files using Adobe Photoshop CS2 (Adobe Systems Inc., Mountain View, CA, USA) and stored on a desktop computer. The images were then imported into AutoCAD 2021 (Autodesk Inc., San Rafael, CA, USA). In AutoCAD, at least thirty points were marked on the tooth outline using the point tool in the Draw Toolbox (Figure 3).

**Figure 4:**
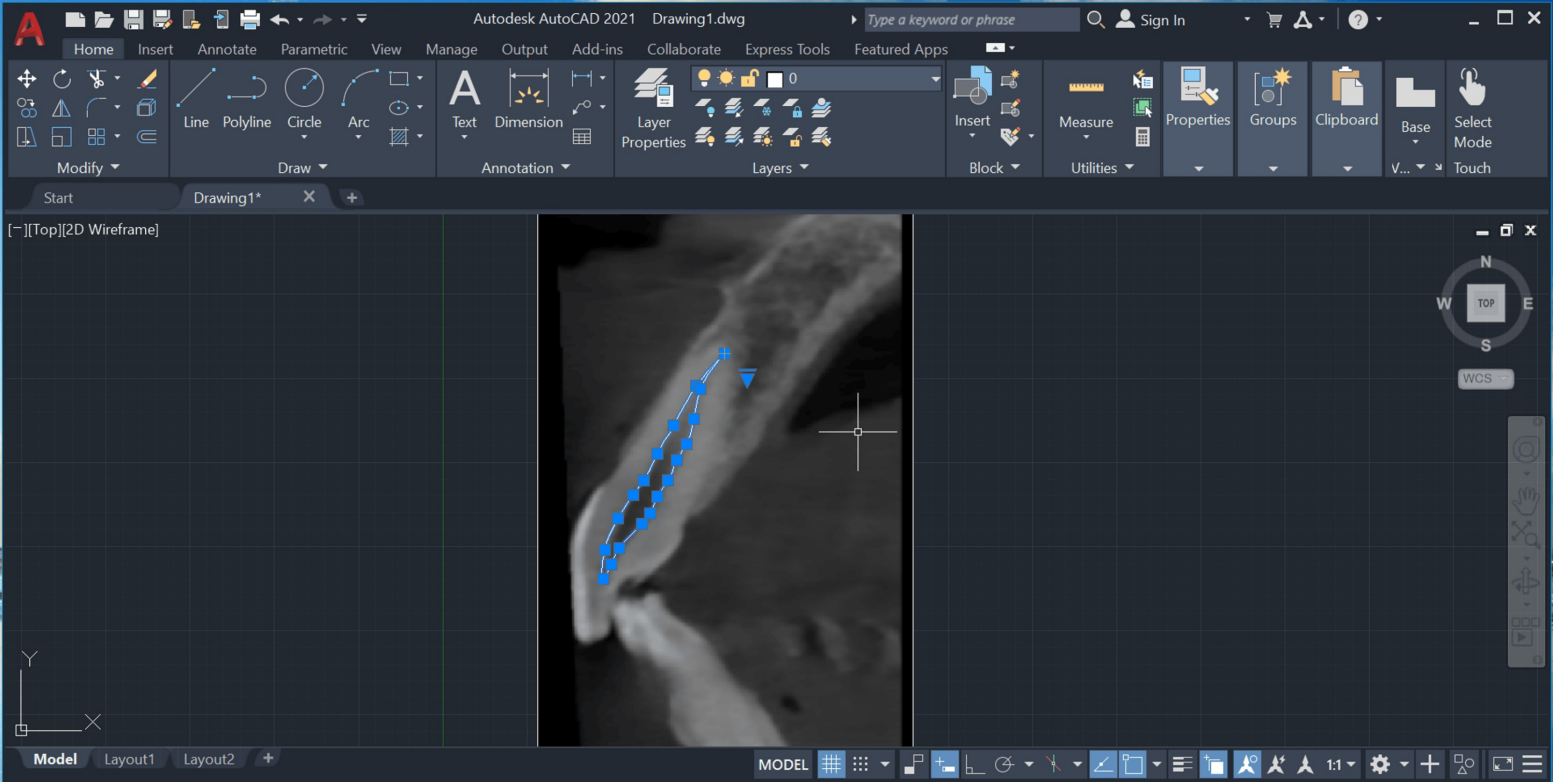
Marking of points on the pulp outline using the point tool on AutoCAD's Draw Toolbox.

Additionally, a minimum of 10 points were marked on the pulp outline and connected using the line tool (Figure 4).

**Figure 5:**
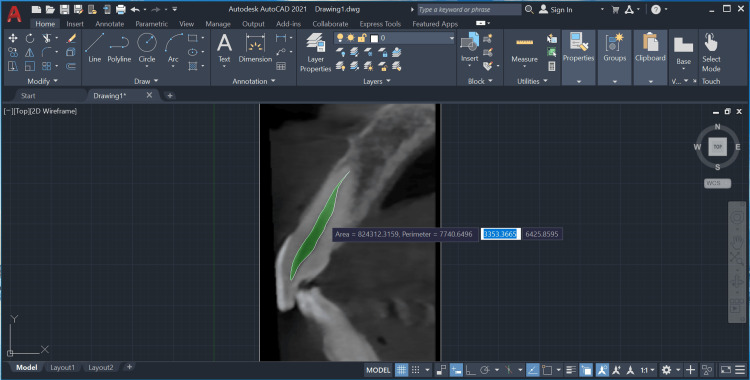
Measurement of the pulp area using the point and line tools on AutoCAD's Draw toolbox.

**Figure 6:**
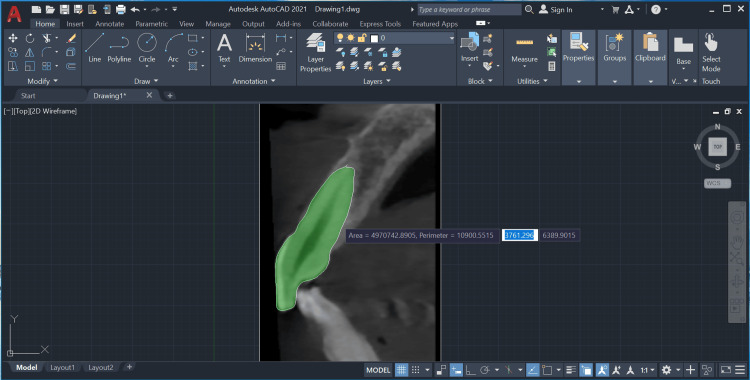
Measurement of the tooth area using the point and line tools on AutoCAD's Draw toolbox.

The pulp and tooth areas were measured with the point and line tools, and the pulp-to-tooth area ratio was calculated (Figures 5, 6).

Data entry

The pulp-to-tooth area ratios and patient demographic data were entered in a Microsoft Excel 2019 (Microsoft Corp., Redmond, WA, USA) sheet. The obtained ratios were utilised for subsequent statistical analysis for which statistical tools and software were utilised.

Statistical data analysis

The data was analysed using IBM SPSS Statistics for Windows, version 23 (IBM Corp., Armonk, NY, USA). The level of significance was kept at 5%. Data was subjected to normality assessment using the Shapiro-Wilk test. Parametric tests were applied for normally distributed data and non-parametric tests for the data did not follow a normal distribution. The penetration depth in each group was presented using mean and standard deviation. Demographic details were presented using descriptive statistics. The correlation between age and pulp-to-tooth area ratio was analysed using either the Pearson correlation test or Spearman's correlation test. The association of age with the pulp-to-tooth area ratio was tested using Linear regression. The correlation between actual age and estimated age was analysed using the Pearson correlation test. The intraclass correlation coefficient (ICC) was used as a measure of intra-examiner and inter-examiner agreement.

## Results

The present study analysed 360 CBCT images of maxillary teeth, including 90 maxillary central incisors, 90 maxillary lateral incisors, 90 maxillary canine and 90 maxillary first premolars of 90 Indian patients (50 males and 40 females) aged between 18 and 70 years, with a mean age of 37.4 years. The intraclass and interclass correlation coefficient (ICC) values revealed good reproducibility of the volumetric measurements. The mean pulp volumes obtained by the inter-examiner and intra-examiner were used for linear regression and Pearson’s correlation analysis.

The study included a total of 40 females and 50 males. The age distribution of the subjects was as follows: eight subjects (8.8%) were under 20 years old, 25 subjects (27.7%) were between 21-30 years old, 23 subjects (25.5%) were between 31-40 years old, 19 subjects (21.1%) were between 41-50 years old, 12 subjects (13.3%) were between 51-60 years old, and three subjects (3.3%) were over 60 years old, as depicted in Table 1.

**Table 1:** Distribution of assessed teeth according to age and sex.

Age group (years)	Male	Female	Total N (%)
<20	5 (62.5%)	3 (37.5%)	8 (8.8%)
21-30	16 (64%)	9 (36%)	25 (27.7%)
31-40	8 (34.7%)	15 (65.2%)	23 (25.5%)
41-50	12 (63.1%)	7 (36.8%)	19 (21.1%)
51-60	7 (58.3%)	5 (41.6%)	12 (13.3%)
>60	2 (66.6%)	1 (33.3%)	3 (3.3%)
Total	50 (55.5%)	40 (44.4%)	90 (100%)

Among the 90 subjects assessed, 57 (63.3%) were evaluated for the right maxillary quadrant, while 33 (36.6%) were evaluated for the left maxillary quadrant, as shown in Table 2. The mean and median values of the pulp-to-tooth area ratio of all assessed teeth are depicted in Table 3. The maximum pulp-to-tooth ratio was seen in the maxillary right first premolar.

**Table 2:** Distribution of assessed teeth according to the maxillary arch quadrant.

Age group (years)	Right side quadrant	Left side quadrant	Total N (%)
<20	3 (37.5%)	5 (62.5%)	8 (8.8%)
21-30	16 (64%)	9 (36%)	25 (27.7%)
31-40	16 (69.5%)	7 (30.4%)	23 (25.5%)
41-50	11 (57.8%)	8 (42.1%)	19 (21.1%)
51-60	8 (66.6%)	4 (33.3%)	12 (13.3%)
>60	3 (100%)	0	3 (3.3%)
Total	57 (63.3%)	33 (36.6%)	90 (100%)

**Table 3:** Mean and median values of the pulp-to-tooth area ratio of all assessed teeth.

Tooth	Number of samples	Mean + standard deviation	Median (min-max)
Maxillary central incisor (right side)	57	0.13 ± 0.03	0.13 (0.04-0.21)
Maxillary lateral incisor (right side)	57	0.17 ± 0.22	0.13 (0.08-1.54)
Maxillary canine (right side)	57	0.16 ± 0.04	0.15 (0.01-0.27)
Maxillary 1st premolar (right side)	57	0.21 ± 0.26	0.17 (0.02-2.09)
Maxillary central incisor (left side)	33	0.14 ± 0.04	0.13 (0.05-0.22)
Maxillary lateral incisor (left side)	33	0.18 ± 0.26	0.13 (0.08-1.64)
Maxillary canine (left side)	33	0.17 ± 0.04	0.16 (0.10-0.26)
Maxillary 1st premolar (left side)	33	0.17 ± 0.05	0.20 (0.02-0.24)

The correlation between actual age and estimated age for each tooth type is shown in Table 4. There was a strong positive correlation between actual age and estimated age for the maxillary right central incisor and maxillary right canine tooth and a weak positive correlation for the maxillary left canine tooth. The p-value in this table indicated a significant correlation with the maxillary right central incisor, maxillary right canine tooth and maxillary left canine.

**Table 4:** Correlation between actual age and estimated age for each tooth type. * indicates a significant correlation at p-value≤0.05.

Tooth type	R-value	p-value
Maxillary central incisor (right side)	0.637	<0.001*
Maxillary lateral incisor (right side)	0.260	0.145
Maxillary canine (right side)	0.405	0.019*
Maxillary 1st premolar (right side)	0.200	0.265
Maxillary central incisor (left side)	0.182	0.176
Maxillary lateral incisor (left side)	0.171	0.204
Maxillary canine (left side)	0.272	0.041*
Maxillary 1st premolar (left side)	0.196	0.144

The linear regression results estimated age based on the pulp-to-tooth area ratio for sex and each type of tooth are depicted in Table 5. There was a strong correlation between age and the pulp-to-tooth area ratio for the maxillary left central incisor and maxillary left canine tooth in males, and a strong correlation between age and the pulp-to-tooth area ratio for the maxillary left central incisor tooth among females. The p-value in this table demonstrated a significant correlation with the maxillary left central incisor and maxillary left canine tooth in males and with the maxillary left central incisor tooth in females.

**Table 5:** Linear regression analysis of estimated age based on the pulp-to-tooth area ratio by sex and tooth type. * indicates a significant correlation at p-value≤0.05. SE - Standard Error This table presents significant correlations between estimated age, sex, and type of tooth.

Tooth type	Male				Female			
	R	R^2^	SE	p-value	R	R^2^	SE	p-value
Maxillary central incisor (right side)	-0.332	0.110	13.758	0.074	0.043	0.002	13.003	0.831
Maxillary lateral incisor (right side)	-0.331	0.109	13.764	0.074	-0.065	0.004	12.988	0.749
Maxillary canine (right side)	-0.417	0.174	13.252	0.550	-0.120	0.014	12.920	0.022*
Maxillary 1st premolar (right side)	-0.079	0.006	14.537	0.677	-0.277	0.077	12.506	0.162
Maxillary central incisor (left side)	-0.627	0.393	10.902	0.003*	-0.647	0.419	9.723	0.017*
Maxillary lateral incisor (left side)	-0.274	0.075	13.461	0.242	-0.476	0.226	11.217	0.100
Maxillary canine (left side)	-0.462	0.214	12.411	0.040*	-0.272	0.074	12.271	0.369
Maxillary 1st premolar (left side)	-0.139	0.019	13.862	0.559	-0.334	0.111	12.020	0.265

## Discussion

Age estimation is a crucial aspect of forensic odontology. Postmortem age estimation using radiography has been extensively employed, particularly by analysing the dimensional changes in the pulp area relative to the tooth area and the resulting ratio. The assessment of the pulp-to-tooth area ratio is an indirect measurement of secondary dentine deposition, which generally correlates well with the subject's chronological age. Secondary dentine, which is surrounded by harder tissues such as enamel, cementum, and primary dentine, is preferred for age estimation [2].

The advent of CBCT offers new opportunities for obtaining 3D views of teeth, providing high-quality images at a low radiation dose compared to CT [8]. CBCT has several advantages over conventional radiographic methods, including controlled magnification, lack of superimposition, absence of geometric distortion, and convenient multiplanar and 3D displays, which enhance structure visualisation and diagnostic accuracy [2].

Considering these observations, the present study was conducted on the maxillary central incisor, lateral incisor, canine, and first premolar teeth in both the right and left quadrants. The results are discussed below.

In the present study, 90 patients were examined, consisting of 50 males and 40 females (Table 1). The participants' ages ranged from 18 to 70 years and were well distributed across different age groups, each spanning a 10-year range.

Similar to the present study, Rai et al. studied 60 patients in 2016, with an equal distribution of 30 males and 30 females [2]. Their ages varied from 20 to 85 years and were evenly spread across distinct age groups, each covering 10 years [2]. In 2018, Gulsahi et al. examined 204 patients, with 108 males and 96 females [9]. The age range for participants was 15 to 70 years, and their distribution across age groups was uniform, with each spanning a 10-year interval [9]. Likewise, Salemi et al. investigated 300 patients in 2020, encompassing 142 females and 158 males aged between 14 and 60 years [3]. The participants' ages were also evenly distributed across different age groups, each covering a 10-year range [3].

In contrast to the present study, in 2021, Pires et al. examined 90 patients with 360 teeth, aged between 18 and 70 years, with 50 males and 40 females [10]. The participant's ages were evenly distributed into six different age groups, each covering a nine-year range [10]. In 2019, Ravipati and Guttikonda conducted a study with 200 patients of whom 114 were males and 86 were females [11]. The participants' ages ranged from 10 to 70 years and were evenly divided into three different age groups, each spanning 20 years [11]. In 2020, Elgazzar et al. conducted a study that comprised 200 CBCT images, consisting of 98 males and 102 females, with chronological ages ranging from 15 to 60 years [7]. The participant's ages were evenly distributed across different age groups, with each age group spanning a four-year range [7]. The distribution of age groups in their studies, with each group covering a specific age range, differs from the present study.

In the present study, both the right and left quadrants of the maxilla were analysed. Out of the 90 patients, 57 patients were assessed for the right side, while 33 were assessed for the left side (Table 2).

In 2013, Ahmed et al. conducted a study involving forty-eight cases, with an equal distribution of 24 males and 24 females [12]. The study entailed multiple measurements on the right maxillary central incisor, right maxillary lateral incisors, right maxillary canine, and right first premolar teeth. Unlike the present study, which includes both right and left-side quadrants, the study by Ahmed et al focused solely on the right-side quadrant [12]. Despite the similar tooth types examined, the present study differs in its comprehensive analysis of both sides of the quadrant [12]. On the other hand, the 2020 study by Zhan et al. utilised 392 CBCT images from individuals spanning various ages and genders [13]. Their focus was on assessing the left maxillary central incisor, lateral incisor, and canine teeth [13].

In the present study, among all types of teeth examined, the maxillary right first premolar showed the highest mean value of the pulp-to-tooth ratio, whereas the maxillary right central incisor tooth displayed the lowest mean value of the pulp-to-tooth ratio (Table 3). In the present study, it was observed that, contrary to expectations, the maxillary central incisor had a lower pulp-to-tooth area ratio compared to the maxillary lateral incisor, which is usually expected to have the lowest ratio. This unexpected result could be attributed to potential variations in the methodology used to measure the pulp-to-tooth area ratio. Inconsistencies or inaccuracies in imaging techniques such as radiographs or CBCT scans, as well as issues in the analysis process, might influence the present study findings. Additionally, the size and composition of the present study sample, including factors like age and gender distribution, could have impacted the results. A smaller sample size could potentially result in atypical ratios being observed.

In 2013, Cameriere et al. conducted a study using peri-apical X-rays of 427 lateral and central incisors, with a sample distribution of 19% maxillary central incisors, 26% maxillary lateral incisors, 28% mandibular central incisors, and 27% mandibular lateral incisors [14]. Of these, 43.3% belonged to women and 56.7% to men. The study found that age estimation, based on age-related changes in the pulp/tooth area ratio, was more accurate when analysing maxillary lateral incisors [14]. Despite this, although the study by Cameriere et al. suggests that the lateral incisor is more reliable for age estimation, most other studies, such as those by Andrade et al., Uğur Aydın et al., Asif et al., and Haghanifar et al., indicate that the maxillary central incisor is generally considered more reliable [15,16,17,18]. The present study findings align with those supporting the maxillary central incisor's reliability for age estimation, indicating potential methodological and sample-related factors that need to be considered in future research.

In the 2018 study by Gulsahi et al., the sample comprised 108 males (52.9%) and 96 females (47.1%) [9]. The study encompassed 91 maxillary central incisors, 102 maxillary lateral incisors, 113 maxillary canines, 131 mandibular canines, 125 mandibular first premolars, and 93 mandibular second premolars. Contrary to the present study, the research by Gulsahi et al. revealed that the mandibular first premolar and maxillary canine exhibited the highest mean value of pulp-to-tooth ratio, while the maxillary lateral incisor displayed the lowest mean value of pulp-to-tooth ratio [9].

The mean age is a frequently used metric in numerous research studies, offering valuable insights into the typical age of the participants. In the present study, 90 samples were analysed, with ages ranging from 18 to 70 years, a mean age of 37.4 years, and a standard deviation of 12.9. In a study conducted by Akbar et al. in 2018, 50 samples were analysed, ranging from 20 to 50 years old, with a mean age of 30.3 years and a standard deviation of 9.45 [1]. In another study conducted by Molina et al. in 2021, a total of 107 patients were examined, involving 313 teeth, including incisors, canines, and mandibular premolars [19]. Out of these patients, 56 were female and 51 were male, with an average age of 44 ± 14 years and an age range of 14 to 70 years [19].

In the present study, we found a strong positive correlation between actual age and estimated age for the maxillary right central incisor and maxillary right canine tooth, and a weak positive correlation for the maxillary left canine tooth. The p-value in this table indicated a significant correlation for the maxillary right central incisor, maxillary right canine tooth, and maxillary left canine tooth (Table 4).

Similar findings were observed in previous studies. In 2011, an investigation by Zaher et al. revealed a statistically significant correlation between age and the pulp-to-tooth area ratio for maxillary central and lateral incisors, suggesting their reliability in estimating age in Egyptians for forensic purposes [20]. In 2019, Andrade et al. investigated the relationship between chronological age and pulp volume, a negative and statistically significant correlation for both types of teeth, with slightly higher coefficients observed for the maxillary central incisor compared to the maxillary canine [15]. Additionally, in 2019, Uğur Aydin et al. conducted a study on the pulp-to-tooth ratio of maxillary central incisor teeth, finding a significant negative correlation between chronological age and pulp-to-tooth area ratio (PTR), indicating its reliability in age estimation within the Turkish population [16].

In 2019, Asif et al. conducted a study on volumetric analysis of the pulp-tooth ratio in maxillary left canines, maxillary right canines, and maxillary right central incisors [17]. Their findings indicated the strongest coefficient of correlation values for maxillary right central incisors, followed by maxillary right canines and maxillary left canines [17]. Similarly, in 2019, Haghanifar et al. conducted a study on measuring PTR in maxillary and mandibular canines and central incisors [18]. Their regression analyses demonstrated that maxillary central incisors were the most reliable for age estimation among the teeth studied, while maxillary canine teeth exhibited the lowest predictive power for estimating age. The studies showed results that closely mirrored those found in the present study [18].

Conversely, in 2013, Cameriere et al. conducted a study investigating peri-apical X-ray measurements of both maxillary and mandibular incisors to examine the relationship between age and age-related changes in the pulp-tooth area ratio [14]. The findings revealed that when age estimation was based on these changes, the maxillary lateral incisors offered more accurate estimations [14].

The present study found a strong correlation between age and the pulp-to-tooth area ratio for both the maxillary left central incisor and maxillary left canine tooth in males and a strong correlation for the maxillary left central incisor tooth in females. The p-value in this table demonstrated a significant correlation for the maxillary left central incisor and maxillary left canine tooth in males, and for the maxillary left central incisor tooth in females. In the present study, was observed that more teeth were significant for age estimation in males compared to females (Table 5).

Conversely, in the study by Someda et al., they observed that the relationship between the pulp-tooth volume ratio and age was stronger for women than for men, although this difference was not statistically significant [21]. Similarly, in the study by Star et al., the relationship between the pulp-tooth volume ratio and age was also stronger for women than for men, but the difference was not statistically significant [22]. Additionally, there was no significant interaction between tooth types and gender [22].

Limitations

The segmentation process was challenging due to its reliance on grayscale values and the inherent resolution of CBCT [23]. The first premolar was included in the study, but most research had not focused on using this tooth for age estimation. More studies in the future may be needed to explore the potential of this tooth [24]. Most studies using CBCT for age estimation measured both pulp and tooth volumes. However, the CBCT software used in this study did not support volumetric measurement, so we only measured the pulp and tooth areas [25]. In this study, there was a lack of access to micro-CT for evaluating the segmentation accuracy of CBCT [26]. The study was hindered by a shortage of retrospective data from older individuals, resulting in a smaller sample size for the older people age group, which could potentially pose a significant limitation [27].

Additional research involving a larger and more diverse set of populations, including various ethnic groups, is necessary for improving dental age estimation through cone beam computed tomography (CBCT) images. This research could utilise different volumetric analysis software to measure pulp and tooth volumes more accurately, thereby enhancing the precision of dental age estimation [18].

## Conclusions

The current study concluded that using the pulp-to-tooth area ratio with cone-beam computerised tomography (CBCT) for age estimation is effective. The correlation between actual and estimated ages varied among different tooth types. It was found that the estimated age for teeth in the right-side quadrant (maxillary central incisor, lateral incisor, canine, and first premolar) was one year higher than the chronological age. In contrast, for the left-side quadrant (maxillary central incisor, lateral incisor, canine, and first premolar), the estimated age was two years lower than the chronological age. A strong positive correlation was noted for the maxillary right central incisor and maxillary right canine, while the maxillary left canine showed a slightly weaker but still positive correlation. This indicates that the maxillary right central incisor and maxillary right canine are the most reliable for age estimation. The study also examined linear regression results for estimating age based on the pulp-to-tooth area ratio, categorized by sex and tooth type. Significant associations between age and the ratio were found for the maxillary left central incisor and maxillary left canine in males, and for the maxillary left central incisor in females.
